# HOXC-AS1 and EZH2 interaction increase HOXC9 expression and promote the malignant transformation of oral leukoplakia

**DOI:** 10.7150/jca.103482

**Published:** 2025-01-13

**Authors:** Xiaochuan Chen, Jiusong Han, Shuhua Li, Xi Yang, Shuyu Yang, Chenrong Xu, Xueyi Liang

**Affiliations:** 1 Stomatological Hospital, School of Stomatology, Southern Medical University, S366 Jiangnan Boulevard, Guangzhou, Guangdong 510280, China.; 2 Guanghua School of Stomatology, Sun Yat-sen University, No. 55 Linyuan Xi Road, Guangzhou, Guangdong 510055, China.

**Keywords:** HOXC-AS1, HOXC9, cancer stem-like cells, oral leukoplakia, oral squamous cell carcinoma

## Abstract

**Objective:** To investigate the role of HOXC9 in the transformation of oral leukoplakia (OLK) to oral squamous cell carcinoma (OSCC) and its effectiveness as a new molecular marker for oral leukoplakia carcinogenesis.

**Materials and Methods**: We assessed HOXC9 in OLK and OSCC using immunohistochemistry (IHC). Colony formation and transwell experiment were employed to appraise the function of HOXC9 in the malignant transformation of OLK. ChIP-qPCR, CO-IP, RIP-qPCR, RNA pull down and mass spectrometry were using to evaluate the molecular mechanism of HOXC9.

**Results:** HOXC9 expression was higher in patients with OSCC than in those with OLK, which is associated with increased malignant transformation of OLK. Functional experiments suggested that HOXC9 induces the acquisition of cancer stem cells (CSCs) and epithelial-to-mesenchymal transition (EMT). Subsequently, we found that the HOXC9-mediated malignant phenotype was reversed by HOXC-AS1 depletion. Mechanistically, HOXC-AS1 regulates H3K27me3 methylation and EZH2 as a potential HOXC-AS1-HOXC9 interacting protein. Finally, we found that the 251-619nt nucleotide of HOXC-AS1 competitively binds to EZH2.

**Conclusion:** HOXC-AS1 competitively binds to EZH2, inhibiting its binding to H3 in the HOXC9 promoter region, resulting in a decrease in H3K27me3 and enhanced expression of HOXC9, thereby promoting CSCs and EMT in oral leukoplakia, ultimately leading to malignant transformation into oral squamous cell carcinoma.

## Introduction

Oral leukoplakia (OLK) can evolve into oral squamous cell carcinoma (OSCC), which has high malignancy and low survival rates 1, 2. A recent meta-analysis of 32 studies reported an estimated overall mean proportion of malignant transformation (MT) of 9.3% in OLK 3. Given the complex clinical scenario associated with OSCC management, it is crucial to clarify the occurrence and development mechanism of OLK and establish effective screening methodologies to identify oral potentially malignant disorders (OPMDs) with higher efficacy, and thus improve the prognosis of patients.

HOXC9, a member of the HOX family, is highly expressed in various tumors such as colorectal cancer, gastric cancer 4 and is related to tumor malignancy and an increased risk of distant metastasis, and is thus an indicator of poor prognosis 5. Recent studies have found that high expression of HOXC9 induces EMT in a variety of cancer cell lines 4, promotes the motility of tumor cells and enhances their self-renewal capabilities 6. Sequencing studies have found that the expression of HOXC9 is abnormally elevated in head and neck squamous cell carcinoma 7. However, whether HOXC9 is closely related to OLK progression is unclear, and the role of HOXC9 in malignant transformation of OLK has not been reported previously.

Previous studies have shown that the occurrence of OLK involves complex molecular biological processes, including abnormal proliferation of epithelial cells acquisition of cell stemness and EMT 8. The study found that the expression of NANOG in the moderate OLK group was significantly lower than that in the severe OLK group, suggesting that NANOG may play a role in the malignant transformation of OLK from mild to severe dysplasia 9. The expression of SOX-2 in the basal and upper basal layers of the OLK group was higher than that in the normal oral mucosa group, and the expression in the upper basal layer was even higher. This suggests that there is an ectopic expression pattern of stem cell like population extending from the basal layer to the upper basal layer during the occurrence of OLK, which seems to be a reliable indicator for distinguishing inert and high-risk cases 10, 11. EMT plays a significant role in promoting the OLK malignant transformation. As an important regulator of EMT, SNAI2 could be a target to block the OLK malignant transformation 12, 13. In summary, the expression of stem cell markers and EMT may play an important role in the occurrence of OLK and indicate extremely high malignant potential.

To further investigate the effect of HOXC9 on the malignant transformation of OLK, we designed overexpressed the HOXC9 sequence to study its role in dysplastic oral keratinocytes (DOK), and designed knocked down HOXC9 with two siRNA sequences to study its role in HSC3 *in vitro*. We also used Co-IP and liquid chromatography-mass spectrometry to identify the potential mechanism of HOXC9 expression, thereby providing a theoretical basis for HOXC9 as a potential therapeutic target for OLK.

## Material and Methods

### DOK and HSC3 cell lines

DOK cells were purchased from Luzhen Industrial Co., Ltd (Shanghai, China), and squamous cell carcinoma (HSC3) cells were purchased from ATCC (Rockville, MD, USA). The cell lines were authenticated by short tandem repeat (STR) analysis using either ATCC or IDEXX Radil. DOK and HSC3 was maintained in DMEM containing 10% fetal bovine serum (FBS), 100 U/mL penicillin and 100 mg/mL streptomycin.

### Immunohistochemistry

Immunohistochemistry was performed as previously described 13. Information of the primary antibodies is listed in Supplementary Table 1. Positive cells were defined as the typical immunostaining. The staining index (SI) for HOXC9/CD133 was scored according to the staining intensity (0, no staining; 1, weak, light yellow; 2, moderate, yellow brown; 3, strong, brown) and the proportion of positive cells (0, negative; 1, < 10%; 2, < 50%; 3, < 75%; 4, ≥75%) according to the following formula: SI = the proportion of positively stained cells + the staining intensity. Inclusion criteria and exclusion criteria for normal oral mucosal epithelium, oral leukoplakia or oral squamous cell carcinoma tissue is listed in Supplementary Table 2.

### RNA pull down and mass spectrometry

Biotin-labeled oligonucleotide probes targeting the junction sites of HOXC-AS1 were synthesized by Jisai Biotechnology Co. Ltd. (Guangzhou, China). HSC3 cell lysates were incubated with the biotinylated probes, followed by incubation with streptavidin-coated magnetic beads to pull down the RNA-protein complex. The retrieved proteins in the complex were analyzed by western blotting or mass spectrometry (Zhuanyan Biotechnology Co., Ltd., Guangzhou, China).

### RNA-binding protein immunoprecipitation

Complete RNA-binding protein immunoprecipitation (RIP) lysate buffer was prepared using the Magna RIP RNA-Binding Protein Immunoprecipitation Kit (17-700; Millipore, USA) as described previously 14.

### Chromatin immunoprecipitation assay

ChIP assays were performed using a ChIP Assay Kit (Millipore, USA) as described previously 15. The primer sequences used for ChIP-qPCR are listed in Supplementary Table 3.

### Co-immunoprecipitation

Whole-cell protein lysates were obtained using IP lysis buffer (1.0% NP-40 lysis buffer, 0.2 mM EDTA, 20 mM pH 8.0 Tris-HCl, 180 mM NaCl), phosphatase inhibitors (Thermo), and protease inhibitor cocktail (Roche) at 4 ºC. After centrifugation at 12,000 rpm for 10 min at 4 ºC, the supernatants were prepared for endogenous IP and incubated with corresponding antibodies and Protein A/G Magnetic Beads (MCE) at 4 ºC overnight. Subsequently, the samples were separated using a magnetic separator, denatured with SDS loading buffer, and examined by western blotting or mass spectrometry.

### Statistical analysis

GraphPad Prism 10.1.2 software (La Jolla, CA, USA) was used for all statistical analysis. All data are presented as means ± standard deviations for at least triplicate samples, unless stated otherwise. Statistical significance was analyzed using Pearson's correlation test, Student's t-test, or Tukey's test after ANOVA, as appropriate. A *P*-value of < 0.05 was considered to reflect a statistically significant difference.

## Results

### The expression of HOXC9 increases with the degree of malignancy of oral epithelium

**Figure 1:**
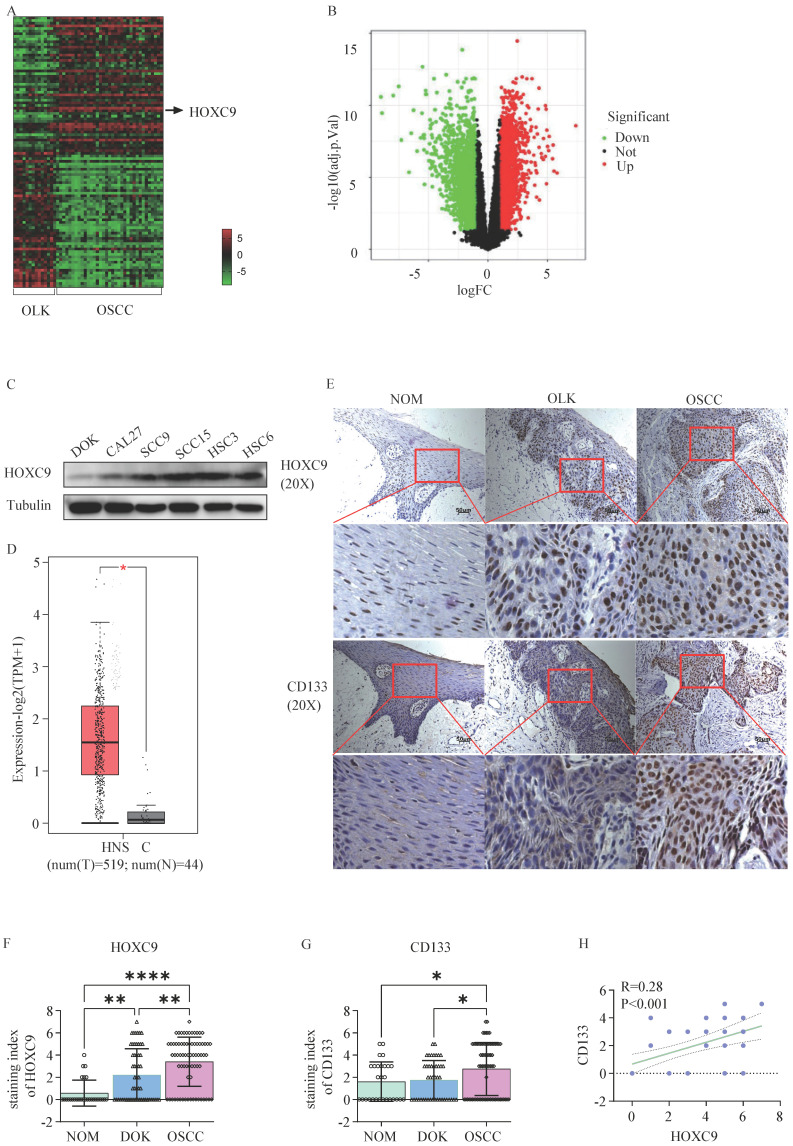
** The HOXC9 expression level increases with the degree of malignancy of oral epithelium.** A. Heatmap of different RNA (up-regulated or down-regulated more than 3-fold) at the transcriptional level from 15 OLK and 34 OSCC tissues based on GEO database (GSE85195). B. Volcano plot of differentially expressed genes in GSE85195. C. The expression of HOXC9 in 5 OSCC cell (CAL27, SCC-9, SCC-15, HSC-3 and HSC-6) and DOK cell was validated by Weston blot analysis. D. The expression of HOXC9 in HNSC tissues and normal tissues in GEPIA based on TCGA database (tumors tissues:519, normal tissues: 44). E. Representative histochemistry images for HOXC9 and CD133 staining in human NOM、OLK and OSCC tissues are shown. F-G. The staining index of HOXC9 or CD133 expression in human NOM, OLK and OSCC tissues. H. Correlation analysis between HOXC9 and CD133 expression in the human OLK tissue. *P < 0.05; **P < 0.001; ***P < 0.0001.

First, we performed differential expression gene analysis in human 34 OSCC and 15 OLK tissues using the GEO database (GSE85195) and presented the results through heatmap (Fig. 1A) and volcano plot (Fig. 1B), In total, we identified 247 upregulated and 171 downregulated genes (fold change >3). Among them, HOXC9 was one of the most differentially expressed mRNAs, and its expression was high in the five OSCC cell lines compare to DOK cells (Fig. 1C). To clarify the HOXC9 expression, we then used GEPIA database (http://gepia.cancer-pku.cn/), which is based on (The Cancer Genome Atlas) TCGA database, to obtain and analyze the relative HOXC9 expression data between head and neck squamous cell carcinoma (HNSCC) tissues and adjacent normal tissues, The results showed that HOXC9 was more highly expressed in HNSCC tissues (Figure 1D). To further confirm the expression of HOXC9, IHC staining was performed in 28 normal oral mucosa(NOM) tissues、45 OLK and 72 OSCC tissues, We found that the HOXC9 expression were enhanced significantly in OLK tissues than in NAT, Meanwhile, The HOXC9 expression in OSCC tissues, were also most highly expressed compared with the NOM and OLK tissues (Fig. 1E), According to statistical analysis, the number of cases with high HOXC9 expression (score > 4 points as high expression) was 0.00% in NOM tissues, 31.11% in OLK tissues, and 40.28% in OSCC tissues (Fig. 1F). We also performed immunohistochemical analysis of CD133, which is a critical markers of cell stemness. As shown in Fig. 1E and Fig. 1G, NOM cases showed lower CD133 immunolabeling when compared with the OLK cases, although this difference was not statistically significant. However, the means were statistically higher in OLK when compared with that in NOM, and in OSCC, the means were statistically highest. According to statistical analysis, the number of cases with high CD133 expression (score > 4 points as high expression) was 3.50% in NOM tissue, 4.44% in OLK tissue, and as high as 34.72% in OSCC tissue (Fig. 1G). In addition, we performed a correlation analysis of HOXC9 and CD133 expression in OLK tissues, and the results showed that there was a positive association between HOXC9 and CD133(Fig. 1H). The above results indicate that the expression of HOXC9 and CD133 increases with the degree of malignancy of oral epithelium and the HOXC9 expression is accompanied by an increase in CD133 expression. However, it is currently unclear whether HOXC9 can promote the malignant transformation of oral leukoplakia by increasing the cell stemness of oral leukoplakia.

### HOXC9 functions as an oncogene in oral leukoplakia

**Figure 2:**
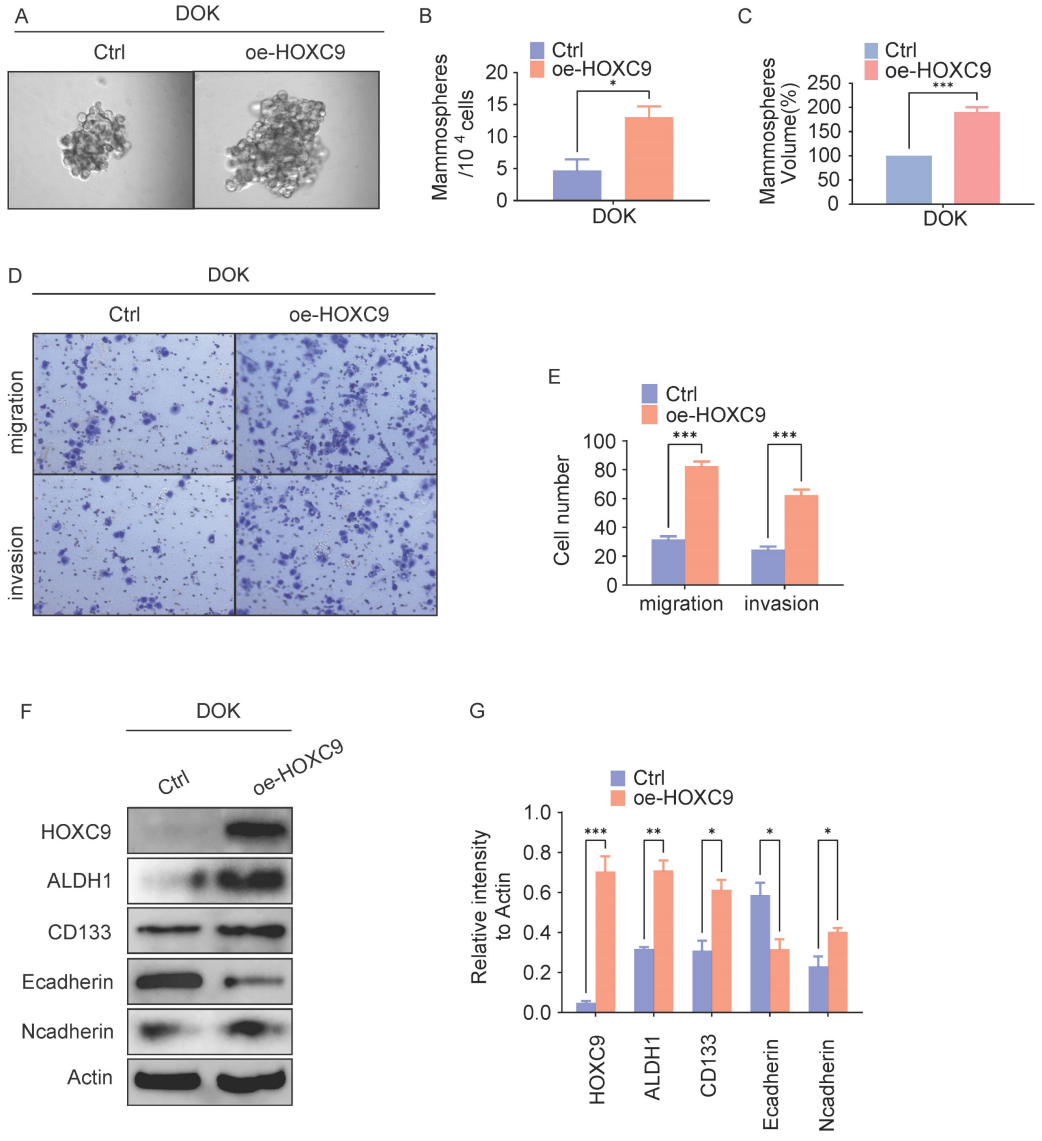
** Overexpression of HOXC9 promotes the self-renewal and epithelial-to-mesenchymal transition of DOK.** F. DOK cells transfected with an empty plasmid or a vector encoding oe-HOXC9 for 48 hours. A-C. Representative micrographs of tumorsphere formation(A), number(B) and size(C) of sphere. D-E. Representative migratory, invasion images (D) and quantification (E) of the migratory, invasion cells. F-G. Western blot was used for analyzing the expression of the EMT-associated proteins: E-cadherin, N-cadherin or CSCs-associated proteins: ALDH1A1, CD133 (F) and the relative intensity to the Actin internal control are shown (G). All the reactions were run in triplicate. *P < 0.05; **P < 0.001; ***P < 0.0001.

To investigate the potential role of the HOXC9 on OLK malignant transformation, we transduced Dysplastic Oral Keratinocyt (DOK) cell which is pre-cancer cell with a HOXC9-overexpressing vector and determined the self-renewal ability reflecting the cancer stem(-like) cells (CSCs) signature. *In vitro* tumorsphere formation analyses demonstrated that HOXC9-overexpressing DOK cells exhibited increased tumorsphere numbers and size when compared to control groups (Fig. 2A-C), Consistently, western blot analysis demonstrated that the expression of CSCs markers (ALDH1A1, CD133) were increased in HOXC9 overexpression groups (Fig. 2F-G), suggesting that upregulated HOXC9 augmented the self-renewal ability of DOK cells.

Given that high self-renewal ability is more invasive and contributes to OLK aggressiveness 11, we next investigated the role of HOXC9 in regulating OLK aggressiveness. *In vitro* transwell invasion analyses demonstrated that overexpression of HOXC9 markedly promotes the number of migrating or invading DOK cells (Fig. 2D-E). As the enhanced invasiveness of DOK is closely linked to their preferential expression of epithelial-mesenchymal transition (EMT) 16, we next examined the levels of EMT markers in DOK. The results demonstrated that the N-cadherin protein levels were increased, while the E-cadherin protein levels were impaired in the HOXC9 overexpression groups (Fig. 2F-G), which proved that overexpression of the HOXC9 gene promotes EMT. Taken together, these results indicate that HOXC9 overexpression correlates with the acquisition of self-renewal ability and invasiveness.

**Figure 3:**
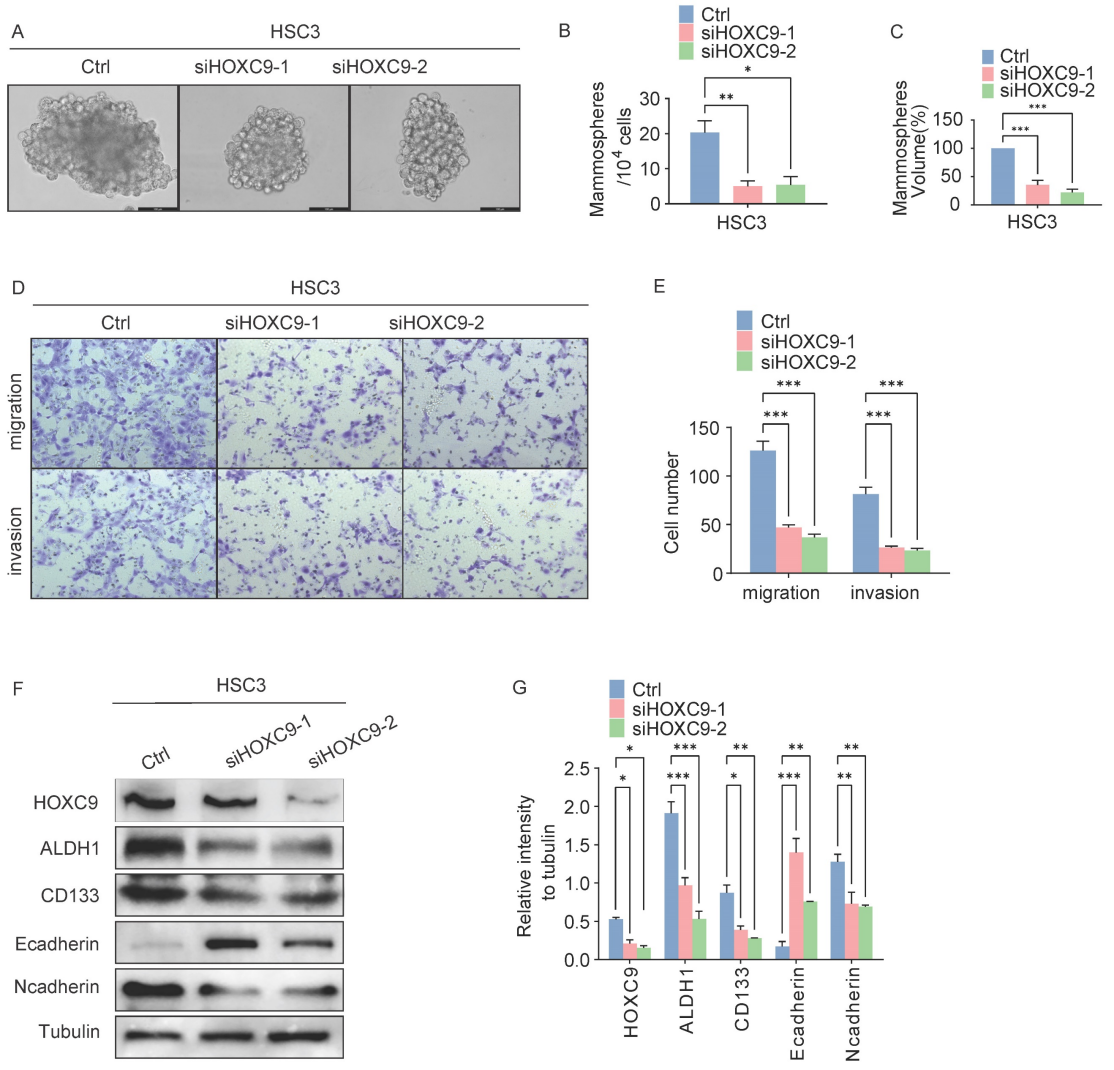
** Disruption of HOXC9 inhibits the self-renewal and epithelial-to-mesenchymal transition of HSC3.** A-F. HSC3 cells transfected with si-NC or si-HOXC9 plasmid for 48 hours. A-C. Representative micrographs of tumorsphere formation (A), number (B) and size (C) of sphere in indicated plasmids. D-E. Representative migratory, invasion images (D) and quantification (E) of the migratory, invasion cells. F-G. Western blot was used for analyzing the expression of the EMT-associated proteins: E-cadherin, N-cadherin or CSCs-associated proteins: ALDH1A1, CD133 (F) and the relative intensity to the tubulin internal control are shown (G). All the reactions were run in triplicate. *P < 0.05; **P < 0.001; ***P < 0.0001.

We further explored the self-renewal ability and invasiveness of HOXC9 in cancerous (HSC3) cells. First, HSC3 cells were infected with siRNA targeting human HOXC9, and the knockdown effect was confirmed by western blotting (Fig. 3F-G), which showed that the knockdown of HOXC9 significantly attenuated the sphere-forming, migratory and invasive ability of HSC3 (Fig. 3A-E). We performed western blot analysis for CSCs and EMT markers in HSC3 cells. N-cadherin, ALDH1A1, and CD133 protein levels decreased, while E-cadherin protein levels were impaired in the HOXC9 knockdown groups (Fig. 3F-G). Taken together, this suggests that HOXC9 knockdown suppressed cancer migration, invasion, and sphere-forming ability in HSC3.

### HOXC-AS1 activates the oncogenic functions of HOXC9 to enhance the self-renewal and EMT ability

**Figure 4:**
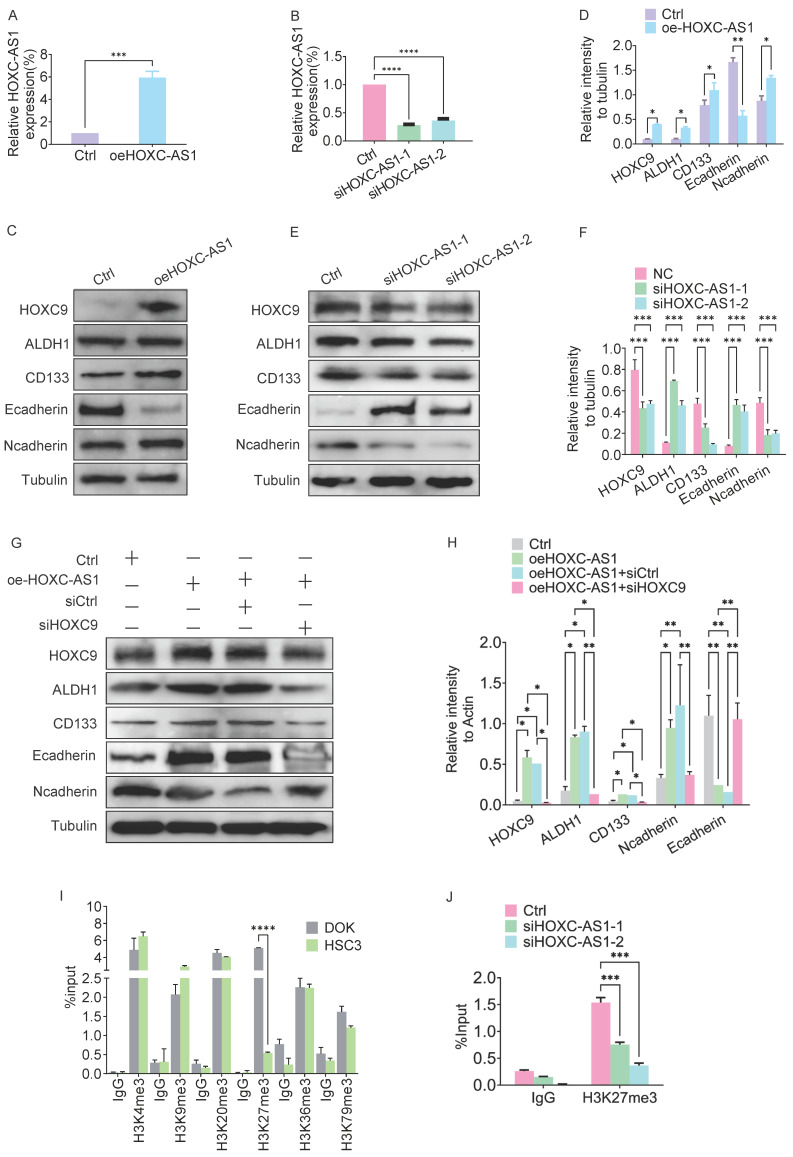
** HOXC-AS1 regulates the EMT and CSCs by regulates H3K27me3 levels in the HOXC9 promoter.** A. q-PCR assay for overexpression efficiency of oeHOXC-AS1 in DOK cells. B. q-PCR assay for knockdown efficiency of siHOXC-AS1-1 and siHOXC-AS1-2 in DOK cells. C-D. Western blot was used for analyzing the protein expressions of E-cadherin, N-cadherin, ALDH1A1, and CD133 in empty vector group or HOXC-AS1 group (C) and the relative intensity to the Tubulin internal control are shown (D). E-F. Western blot was used for analyzing the protein expressions of E-cadherin, N-cadherin, ALDH1A1, and CD133 in siRNA group or si-HOXC-AS1 group (E) and the relative intensity to the Tubulin internal control are shown (F). G-H. Western blot was used for analyzing the protein expressions of HOXC9, ALDH1A1, CD133 Ncadherin and Ecadherin, in empty vector group, HOXC-AS1 group, HOXC-AS1 combining siCtrl group and HOXC-AS1 + si-HOXC9 group, respectively (G) and the relative intensity to the tubulin internal control are shown (H). I. ChIP-qPCR analysis of active methylation marks H3K4me3, H3K9me3, H3K20me3, H3K36me3, and H3K79me3 at HOXC9 gene loci. Compared to the DOK cells, the H3K27me3 level in the HOXC9 promoter region of HSC3 cells was significantly downregulated. J. ChIP-qPCR analysis of H3K27me3 at HOXC9 gene loci after HOXC-AS1-knockdown. All the reactions were run in triplicate. *P < 0.05; **P < 0.001; ***P < 0.0001.

The increased expression of HOXC9 in oral epithelium tissue with increasing malignancy and its promotion of CSCs and EMT indicate a close relationship with the malignant transformation of oral leukoplakia. We focused on the regulatory mechanism of HOXC9 and examined the HOXC9 promoter region in detail using the NCBI website. We identified an antisense long non-coding RNA (lncRNA), HOXC-AS1, upstream of the transcription initiation site (TSS) of HOXC9. We investigated the role of HOXC-AS1 in DOK *in vitro*. After successful transfection of the vector expressing HOXC-AS1 or expressing siRNA targeting HOXC-AS1 in DOK, the overexpression efficiency of HOXC-AS1 (Fig. 4A) or the knockdown efficiency of siHOXC-AS1-1 and siHOXC-AS1-2 (Fig. 4B) was analyzed using q-PCR. the expression level of HOXC9 increased in the HOXC-AS1 overexpression model (Fig. 4C-D) and decreased in the HOXC-AS1-silenced model (Fig. 4E-F). The western blotting results showed that CSCs and EMT markers were enhanced when HOXC-AS1 was overexpressed and inhibited when HOXC-AS1 expression was silenced in DOK cells (Fig. 4E-F). Given these findings, we determined whether HOXC9 is a key downstream effector of HOXC-AS1 that promotes CSCs and EMT. We next performed rescue experiments, which revealed the partial reversal of the inhibitory effects of HOXC-AS1 on tumor sphere formation and EMT in HOXC9 OE DOK cells (Fig. 4G-H). Collectively, these data demonstrated that HOXC-AS1 promotes cell stemness and EMT through HOXC9.

### HOXC-AS1 regulates methylation levels in the HOXC9 promoter region

Given that the HOXC-AS1 coordinate is located in the HOXC9 promoter region, the presence of antisense lncRNAs (HOTTIP and HOTAIR) in the HOX family gene cluster can regulate the expression of HOX genes through epigenetic regulation 17. Therefore, we next investigated the role of HOXC-AS1 in the epigenetic regulation of HOXC9. Because H3 methylation in the HOX gene promoter region is considered a potential biomarker for OSCC 18, we next examined whether HOXC-AS1 regulates HOXC9 mRNA expression by modulating this process. ChIP analysis revealed that compared to DOK, the H3K27me3 level in the HOXC9 promoter region of HSC3 cells was significantly downregulated (Fig. 4I). At the same time, silencing of HOXC-AS1 significantly decreased methylation K27 of histone H3 (H3K27me3) (Fig. 4J). Taken together, these results suggested that HOXC-AS1 promotes the methylation of histone H3 at K27, thereby epigenetically enhancing HOXC9 expression.

### Interaction between HOXC-AS1 and EZH2

**Figure 5:**
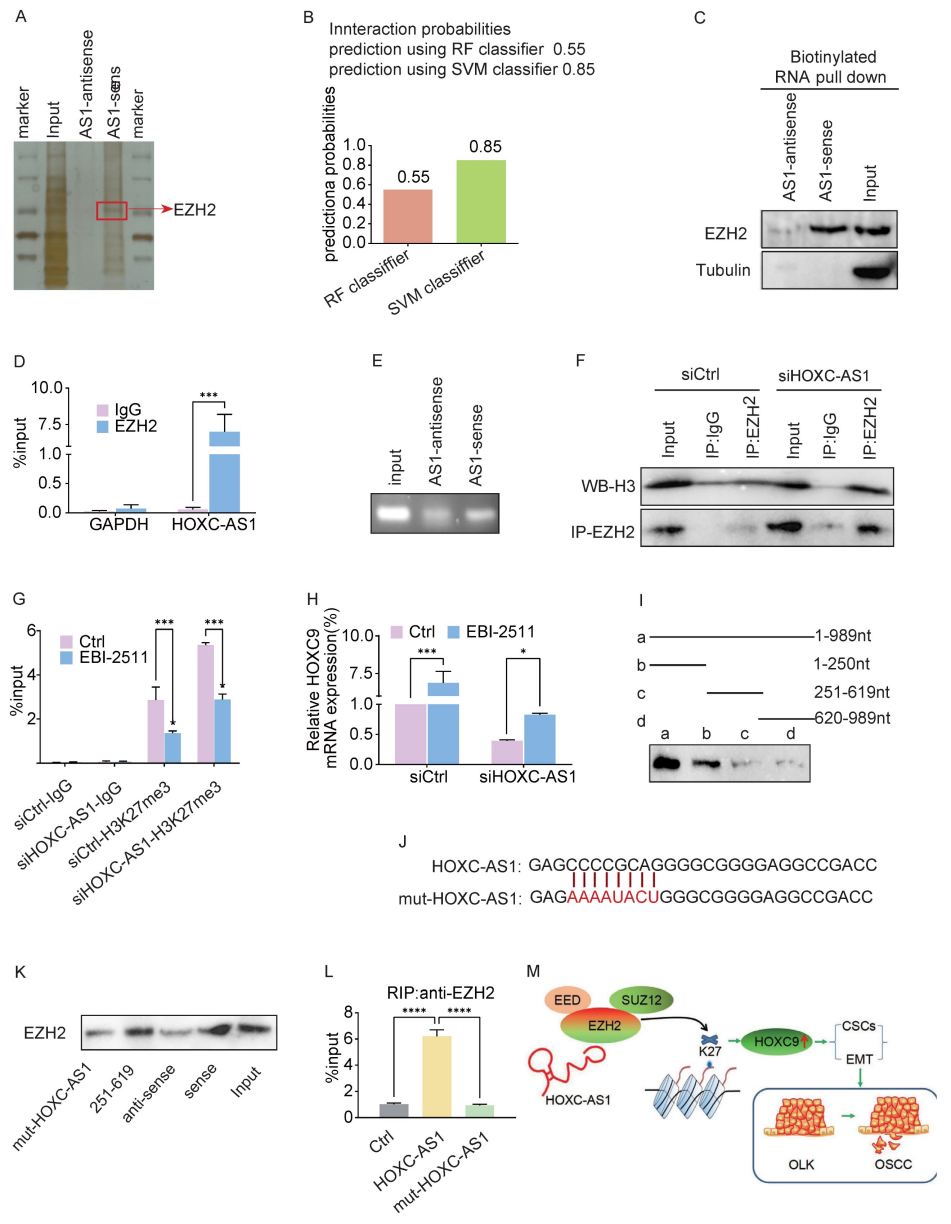
** HOXC-AS1 directly binds with EZH2.** A. Silver staining of proteins by biotinylated sense and antisense HOXC-AS1 pull down with total protein extracts from DOK cells followed by mass spectrum. Among them, EZH2 was one of the potential binding proteins (red arrow). B. Bioinformatic prediction (RNA-Protein Interaction Prediction, RPISeq) was performed to predict the probability of the interaction between HOXC-AS1 and EZH2 by analyzing their sequences. Interaction probabilities generated by RPISeq range from 0 to 1. In performance evaluation experiments, predictions with probabilities >0.5 were considered “positive”. C. Immunoblotting for specific correlation of EZH2 with HOXC-AS1 from RNA pull-down assays. D-E. RNA-binding protein immunoprecipitation (RIP) assay was further used to validate the interaction between EZH2 with HOXC-AS1, followed by qRT-PCR analysis (D) or agarose gel electrophoresis (E). F. Co-immunoprecipitation analysis of the EZH2 and H3 binding after HOXC-AS1-knockdown. G-H. ChIP-qPCR analysis of H3K27me3 at HOXC9 gene loci(G) or q-PCR analysis of HOXC9(H) after DOK cells were transfected with indicated siRNA or plasmids and followed treatment with EBI-2511 or DMSO for 48 hours. I. RNA pull-down assay for the EZH2 binding region of HOXC-AS1 using the truncated mapping. The relevant full-length and truncated mapping. J. Schematic diagram of the mutation strategy. K. Immunoblot showing the EZH2 binding region in 251 to 619 nt of HOXC-AS1. L. The enrichment efficiency of HOXC-AS1 by RIP assays using the EZH2 antibody in HOXC-AS1 OE and mut-HOXC-AS1 cells. (M) The schematic diagram shows the mechanism that HOXC-AS1 regulates HOXC9 expression by competitively binding to EZH2, preventing it from binding to H3 in the HOXC9 promoter region, resulting in a decrease in K27 trimethylation level of H3, which may be the underlying mechanism of the detrimental roles of HOXC9 in OLK progression. All the reactions were run in triplicate. *P < 0.05; **P < 0.001; ***P < 0.0001.

Given that lncRNAs exert their functions mainly by binding to specific proteins 19, we next performed an RNA pull-down assay followed by mass spectrometry to screen for HOXC-AS1-interacting proteins, which revealed that EZH2 was a putative HOXC-AS1 binding protein (Fig. 5A). Zeste homolog 2 enhancer (EZH2), an oncogene that is overexpressed in various human cancers, is a subunit of polycomb inhibitory complex 2 (PRC2), which binds to approximately 20% of lncRNAs and catalyzes H3K27 trimethylase activity through lncRNAs 20, 21. Next, we searched the bioinformatics prediction site RNAInter (http://www.rnainter.org/) and found that EZH2 is a HOXC-AS1-interacting protein (Fig. 5B). In addition, RNA-Protein Interaction Prediction (RPISeq) (http://pridb.gdcb.iastate.edu/RPISeq/) was performed to estimate the interaction probabilities between HOXC-AS1 and EZH2, the results of which confirmed their interaction (Fig. 5B). We performed RNA pull-down assays followed by western blotting to clarify the association between EZH2 and HOXC-AS1. The results were consistent in that EZH2 was pulled down in the biotin-labeled sense HOXC-AS1 group (Fig. 5C). To further confirm this interaction, we conducted an RIP assay followed by agarose gel electrophoresis and qRT-PCR analysis, the results of which supported the enrichment of HOXC-AS1 by EZH2 (Fig. 5D, E). In addition, co-IP analysis showed that endogenous EZH2 interacted with H3 in DOK cells, and that silencing HOXC-AS1 significantly enhanced the binding between EZH2 and H3 (Fig. 5F). To verify whether the regulation of EZH2 by HOXC-AS1 is related to H3K27 trimethylase activity, selective EZH2 inhibitor (EBI-2511) was first added to the DOK to inhibit protein synthesis, while silencing of HOXC-AS1 increased the H3K27me3 level in the HOXC9 promoter region of DOK cells (Fig. 5G) and suppressed the expression of HOXC9 (Fig. 5H). Taken together, these results suggested that HOXC-AS1 acts as a decoy to competitively bind EZH2 and inhibit EZH2-mediated H3K27 trimethylase activity.

To determine which region of HOXC-AS1 is responsible for binding to EZH2, we first employed a truncated mapping strategy using *in vitro* transcribed HOXC-AS1 fragments to pull down EZH2. The data revealed that the second fragment containing 251-619nt was associated with EZH2, similar to the full-length HOXC-AS1 (Fig. 5I), suggesting that 251-619 nt are critical for the interaction of HOXC-AS1 with EZH2. Next, we constructed a mutant (Fig. 5J). Consequently, the mutant completely abolished the binding of HOXC-AS1 with EZH2 (Fig. 2K). Moreover, OE of HOXC-AS1 increased and OE of HOXC-AS1 mutant failed to increase the binding of EZH2 (Fig. 2L). Taken together, these data indicated that HOXC-AS1 physically interacts with EZH2.

### HOXC-AS1 and EZH2 Coordinate CSCs and EMT through HOXC9

**Figure 6:**
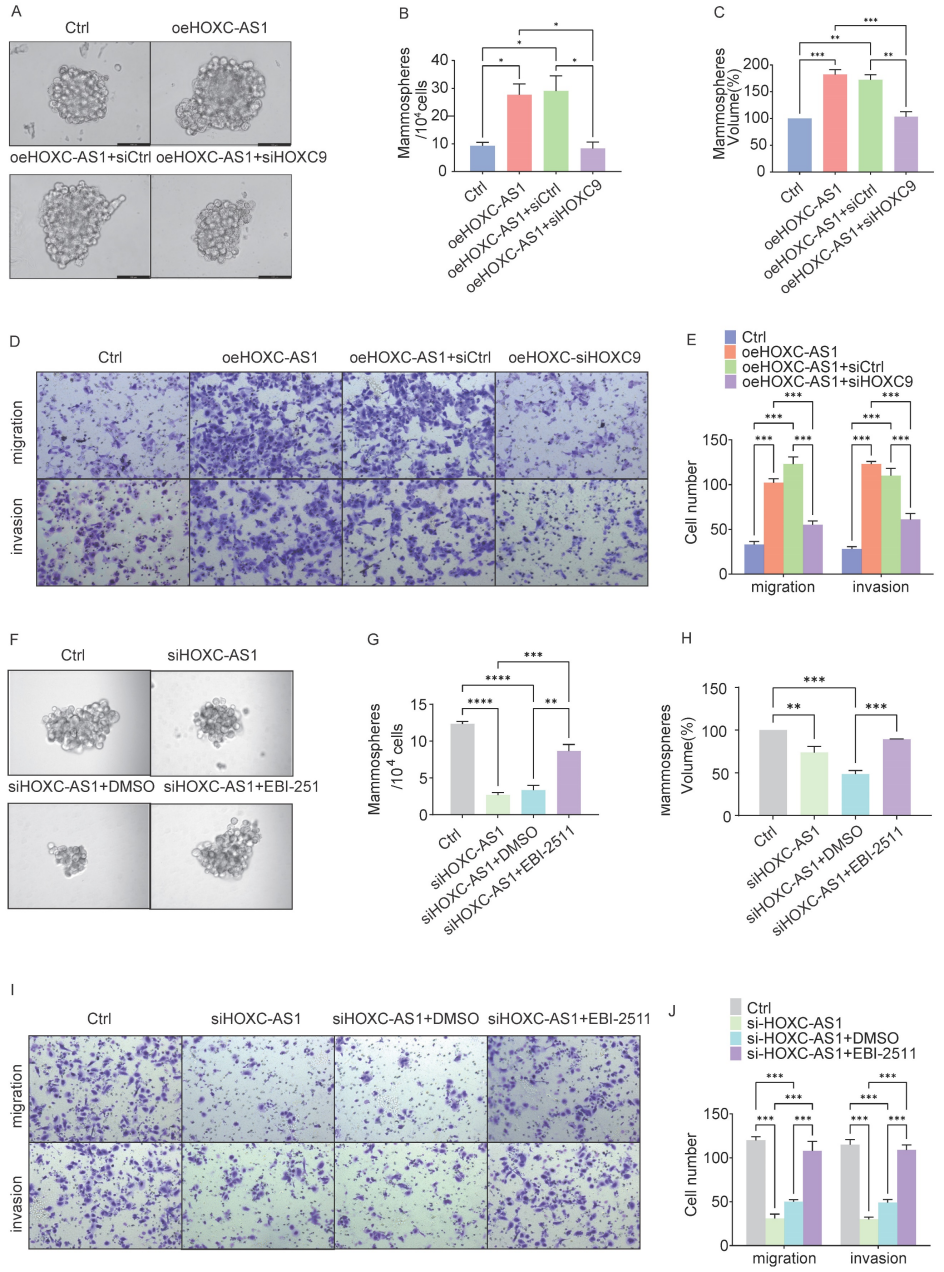
** HOXC-AS1 accelerates cell stem cells, migration, and invasion through elevating the expression of HOXC9.** A-E: DOK cells transfected with the indicated plasmids for 48 hours: A-C. The spheroid formation abilities were examined by sphere formation analysis. Representative tumorsphere formation pictures (A) and the mean number (B) or the mean size (C) of tumorsphere formation are shown. D-E. The migration and invasion abilities were examined by transwell assay. Representative migratory, invasion pictures (D) and the mean number of cells that migrated or invaded to the lower surface are shown (E). F-J: DOK cells transfected with indicated Ctrl or si-HOXC-AS1 plasmids and followed treatment with EBI-2511 or DMSO for 48 hours. F-H. The spheroid formation abilities were examined by Sphere formation analysis. Representative tumorsphere formation pictures (F) and the mean number (G) or the mean size (H) of tumorsphere formation are shown. (I-J) The migration and invasion abilities were examined by Transwell assay. Representative migratory, invasion pictures (I) and the mean number of cells that migrated or invaded to the lower surface are shown (J). All the reactions were run in triplicate. *P < 0.05; **P < 0.001; ***P < 0.0001.

Given these findings, we next investigated whether HOXC9 is a key downstream effector of the HOXC-AS1/EZH2 axis that promotes CSCs and EMT in DOK cells. In rescue experiments, DOK cells were infected with oeHOXC-AS1 lentivirus to overexpress HOXC-AS1 and then infected with siRNA to knockdown HOXC9. The sphere formation analysis showed that the spheroid formation of the oeHOXC-AS1 group increased significantly compared with the Ctrl group, However, knockdown of HOXC9 partly reversed the increased spheroid formation ability caused by HOXC-AS1 overexpression (Fig. 6A-C).

Similar results were obtained in the transwell assay (Fig. 6D-E). The average number of migratory cells per field was 33 in the control group, which, when HOXC-AS1 was overexpressed, increased to 102 (*P*<0.0001), before decreasing to 55(*P*<0.0001) following HOXC-AS1 knockdown. Similar results were observed when the fold change in invasion was calculated. These results indicated that HOXC9 overexpression reversed the increased migration and invasion ability caused by HOXC-AS1 overexpression.

As EBI-2511 is a highly selective EZH2 inhibitor, we next explored the blocking effects of EZH2 release on spheroid formation after HOXC-AS1 depletion. Strikingly, EBI-2511 restored the spheroid formation caused by HOXC-AS1 suppression (Fig. 6F-H). Similar results were observed when the migration or invasion fold-changes were calculated (Fig. 6I-J). These results indicated that EBI-2511 restored the migration and invasion ability of DOK HOXC-AS1 knockdown.

In conclusion, we showed that HOXC9 knockdown can restore the CSCs and migration abilities of DOK cells overexpressing HOXC-AS1, thereby suggesting that HOXC-AS1 interacts with HOXC9 to increase tumor stemness of oral leukoplakia and promote malignant transformation of oral leukoplakia.

## Discussion and Conclusion

Oral leukoplakia can evolve into oral squamous cell carcinoma with high malignancy and a low survival rate 2, therefore, there is an urgent need to identify more effective molecular markers to diagnose oral leukoplakia with potential deterioration. In this study, our results altogether revealed that HOXC9 is upregulated in OSCC cells and tissues. The levels of HOXC9 expression increases with the degree of malignancy of oral epithelium, while knockdown of HOXC9 significantly inhibited CSCs and EMT. These findings demonstrated the oncogenic role of HOXC9 in OSCC.

lncRNAs are emerging as important factors in the malignant progression of tumors such as OSCC 22. Notably, lncRNAs can also interact with RNA-binding proteins by acting as molecular scaffolds and affecting gene expression through post-transcriptional regulation 23. Recently, HOXC-AS1 has been reported to bind to RNA-binding protein-like insulin-like growth factor 2 mRNA-binding protein 2 (IGF2BP2) 24, miR-99a-3p 25, and miR-4651 26 in various tumors, increasing target protein expression, stability, and activity In their study, Ma et al. demonstrated that lncRNA NEAT1 can inhibit GADD45A expression by recruiting BRG1 and installing H3K27me3 and H3K4me3 in the promoter region of GADD45A, thereby promoting gastric cancer progression 27. In this study, we discovered HOXC-AS1 to promote HOXC9 expression by inhibiting H3K27 methylation in its promoter, while knockdown of HOXC-AS1 partially reversed the function of HOXC9 in OSCC CSCs and EMT. This suggests that HOXC9 may be a potential HOXC-AS1-interacting gene, and that further exploration and research are warranted. In addition, how HOXC-AS1 and HOXC9 interact to promote CSCs and EMT in DOK cells requires further investigation.

Due to the positive regulation of the H3K27me3 level by EZH2 that has previously been reported 28, we predicted the potential binding between HOXC-AS1 and EZH2 through the RPISeq website and confirmed their interaction. An oncogene that is overexpressed in various human cancers 28, 29. EZH2 is a subunit of the PRC2 that binds to approximately 20% of lncRNAs and catalyzes the H3K27 trimethylase activity of the EZH2 subunit through lncRNAs, acting as a transcription inhibitor 27. We then explored the critical sequences and domains involved in the interactions between HOXC-AS1, EZH2, and HOXC9. From immunoprecipitation experiments, we found that HOXC-AS1 probably interacted with EZH2, with nucleotides 251-619 of HOXC-AS1 required for binding. In HOXC-AS1-overexpressing OSCC cells, HOXC9 methylation was significantly inhibited and protein stability was upregulated. These results indicated that HOXC-AS1 acts as a decoy by inhibiting HOXC9 methylation and promoting protein stability. Overexpression of EZH2 is related to the inhibition of tumor suppressor and metastasis-related genes and has been proven to have carcinogenic effects on various types of tumors, leading to tumor glycolysis, EMT, migration, and invasion 30. Research has demonstrated that high expression of EZH2 in OSCC is associated with low survival rates in patients 31. EZH2 can determine the expression levels of downstream genes in both directions through both PRC2-dependent transcription regulation and PRC2-independent translation regulation 32, 33; therefore, EZH2 probably plays an oncogenic role in various tumor types, and ubiquitination of EZH2 might be a critical regulatory process in tumorigenesis 34. Following this hypothesis, a rescue experiment indicated that HOXC9 and EZH2 probably act as downstream targets in the process of HOXC-AS1 promotion CSCs and EMT of oral leukoplakia. These results illustrate that HOXC-AS1 promotes malignant transformation of oral leukoplakia by inhibiting the interaction between HOXC9 and EZH2 and the methylation of HOXC9.

This study has some limitations. First, HOXC-AS1 function with HOXC9 deserve further investigation. In this study, pull-down and LC-MS identified 328 proteins that may interact with HOXC-AS1. Additionally, EZH2 is a protein that may interact with HOXC-AS1 to promote CSCs and EMT were selected by bioinformatics. Therefore, we selected only EZH2 proteins for subsequent pull-down verification. The validation results showed that HOXC-AS1 interacts with the target protein EZH2. Proteins other than EZH2 are worthy of further exploration. In addition, the role of HOXC9 in promoting CSCs and EMT has not been identified, and we plan to further investigate the underlying mechanism in future studies.

In summary, HOXC9 overexpression could serve as a pivotal risk factor associated with worse outcomes in OLK, with substantial value for molecular diagnosis and prognostic determination. The most encouraging finding in this study is that we showed for the first time that HOXC-AS1 regulates HOXC9 expression by competitively binding to EZH2, preventing it from binding to H3 in the HOXC9 promoter region, resulting in a decrease in K27 trimethylation level of H3, which may be the underlying mechanism of the detrimental roles of HOXC9 in OLK progression (Fig. 5M). Our study showed that the HOXC-AS1/EZH2/HOXC9 axis plays a key role in the malignant transformation of oral leukoplakia by regulating CSCs and EMT.

## Supplementary Material

Supplementary methods and tables.
