# Supplementary material for: HOXC-AS1 and EZH2 interaction increase HOXC9 expression and promote the malignant transformation of oral leukoplakia
Source: J Cancer. 2025 Jan 13;16(4):1202–14. doi: 10.7150/jca.103482 (PMC11786031; doi:10.7150/jca.103482)
Supplement: Supplementary file 1 — Supplementary methods and tables. [file jcav16p1202s1.pdf]

Material and Methods

Spheroid formation assay

Single cells were plated in low-adhesion 6-well plates and cultured in serum-free DMEM (Gibco) supplemented with N2, 20 ng/mL human recombinant bFGF, and 20 ng/mL EGF (Gibco). After 1–2 weeks of incubation, the number and size of the spheres in each well were counted microscopically.

Cell lysis and western blotting

Following appropriate culturing, proteins were extracted from whole-cell lysates and analyzed by western blotting using polyclonal or monoclonal antibodies. All primary antibodies were used at 1:100–1:1000 dilutions. Full scans of western blots were visualized using chemiluminescent and fluorescent imaging systems. The relative quantities of the proteins were normalized to those of Actin, tubulin. Information regarding the antibodies used in this study is presented in Supplementary Table1.

Real-time quantitative reverse transcription-polymerase chain reaction analysis

Total RNA was extracted from the cells in lysis buffer (Invitrogen) and reverse-transcribed to complementary DNA using the Transcriptor First Strand cDNA Synthesis Kit (Roche) according to the manufacturer's instructions. Real-time quantitative reverse transcription-polymerase chain reaction (qRT-PCR) was performed using SYBR Green I Master Mix (Roche) on a Light Cycler 480 system (Roche), as previously described[6]. All results were normalized to GAPDH expression levels. Information regarding the specific primers used for qRT-PCR is provided in Supplementary Table4.

Plasmid and siRNA transfection

The overexpression plasmid carrying full-length cDNA of human HOXC-AS1 and HOXC9 and their respective negative controls, as well as siRNAs targeting human

HOXC-AS1, HOXC9, and human nonsense control siRNA were obtained from Aiji Biotechnology Co., Ltd. (Guangzhou, China). Cells were transfected with plasmids or siRNAs using Lipofectamine 3000 (Invitrogen) according to the manufacturer's protocol. Information regarding the target sequences of the siRNAs is provided in Supplementary Table5.

Supplementary Table:

Supplementary Table 1: List of antibodies used in current study

Antibody	Company	Product number
ALDH1A1	affinity	BF0220
CD133	affinity	AF5120
E-Cadherin	Cell Signaling	3195
N-Cadherin	Cell Signaling	13116
HOXC9	Abcam	Ab50839
Ezh2	Cell Signaling	2146
H3	Proteintech	68345-1-lg
Tri-Methyl-Histone H3(Lys27)	Cell Signaling	5499S
Tri-Methyl-Histone H3(Lys4)	Cell Signaling	9751T
Tri-Methyl-Histone H3 (Lys9)	Cell Signaling	55286S
Tri-Methyl-Histone H3(Lys36)	Cell Signaling	9763S
Tri-Methyl-Histone H3(Lys79)	Cell Signaling	74073S
GAPDH	affinity	T0004
GAPDH	Cell Signaling	2118L
α-Tubulin	affinity	AF0524
Anti-rabbit IgG, HRP-linked Antibody	Cell Signaling	7074
Anti-mouse IgG, HRP-linked Antibody	Cell Signaling	7076

Supplementary Table 2:List of inclusion criteria and exclusion criteria for normal oral mucosal epithelium, oral leukoplakia or oral squamous cell carcinoma

	inclusion criteria	exclusion criteria
Oral mucosal epithelium	1. 18-65 years old;<br>2. regardless of gender;<br>3. without systemic diseases, and their medical history;<br>4. clinical manifestations and imaging	1. soft tissue diseases such as periodontal disease or oral mucosal disease in gingiva through clinical and imaging examination,

	diagnosis met the WHO diagnostic criteria for impacted teeth.	2. obvious mental disorder;<br>3. pregnant and lactating women.
oral leukoplakia	1. 18-65 years old;<br>2. regardless of gender;<br>3. without systemic diseases, and their medical history;<br>4. clinical manifestations and pathological diagnosis were in line with the WHO diagnostic criteria for oral leukoplakia.	1. previous history of oral leukoplakia;<br>2. obvious mental disorder;<br>3. pregnant and lactating women.
oral squamous cell carcinoma	1. 18-65 years old;<br>2. regardless of gender;<br>3. without systemic diseases, and their medical history;<br>4. clinical manifestations and pathological diagnosis were in line with the WHO diagnostic criteria for oral squamous cell carcinoma.	1. previous history of oral squamous cell carcinoma;<br>2. obvious mental disorder;<br>3. pregnant and lactating women.

Supplementary Table 3: List of CHIP primers used for SYBR Green qRT-PCR

Gene	primer	Primer Sequence (5'→ 3')
Homo-HOXC9	Forward	CACATTCAACAGGCAGCAG
	Reverse	CGCGAGCCCAGAACTTAAC
Homo-GAPDH	Forward	GGCTCCCACCTTTCTCATCC
	Reverse	GGCCATCCACAGTCTGG

Supplementary Table 4: List of primers used for SYBR Green qRT-PCR

Gene	primer	Primer Sequence (5'→ 3')
Homo-HOXC-AS1	Forward	CAACTCCATCTCTGCGACAC
	Reverse	AACAAGCTACTTGCCCACGA
Homo-HOXC9	Forward	CTCGCTCATCTCTCACGACAA
	Reverse	GACGGAAAATCGCTACAGTCC
Homo-GAPDH	Forward	GCACCGTCAAGGCTGACAAC
	Reverse	TGGTGAAGACGCCAGTGGA

Supplementary Table 5:List of the target sequences of the siRNAs

Gene	primer	Primer Sequence (5'→ 3')
hs-HOXC-AS1-si-1	sense	GCUCCUAGCUCAUCUGAGAAGdTdT
	antisense	CUUCUCAGAUGAGCUAGGAGCdTdT
hs-HOXC-AS1-si-2	sense	AGCGAUUGUCAGUUCGGAACGdTdT
	antisense	CGUUCCGAACUGACAAUCGCUdTdT
hs-HOXC-AS1-si-3	sense	GGAUCUCAUACACCUUUAUCAdTdT
	antisense	UGAUAAAGGUGUAUGAGAUCCdTdT
HS-HOXC9-si-1	sense	CCGGGUUCUCAAUCUCACCGAdTdT
	antisense	UCGGUGAGAUUGAGAACCCGGdTdT
HS-HOXC9-si-2	sense	CCGCAGCUACCCGGACUACAUdTdT
	antisense	AUGUAGUCCGGGUAGCUGCGGdTdT
HS-HOXC9-si-3	sense	GUCCGUGGUAUAUCACCCGUAdTdT
	antisense	UACGGGUGAUAUACCACGGACdTdT
NC	sense	UUCUCCGAACGUGUCACGUdTdT
	antisense	ACGUGACACGUUCGGAGAAdTdT
